# Errors introduced by dose scaling for relative dosimetry

**DOI:** 10.1120/jacmp.v13i5.3930

**Published:** 2012-09-06

**Authors:** Yoichi Watanabe, Naoki Hayashi

**Affiliations:** ^1^ Department of Therapeutic Radiology University of Minnesota Minneapolis MN USA; ^2^ School of Health Sciences Fujita Health University Aichi Japan

**Keywords:** relative dosimetry, estimation errors, polymer gel, EDR2, EBT2

## Abstract

Some dosimeters require a relationship between detector signal and delivered dose. The relationship (characteristic curve or calibration equation) usually depends on the environment under which the dosimeters are manufactured or stored. To compensate for the difference in radiation response among different batches of dosimeters, the measured dose can be scaled by normalizing the measured dose to a specific dose. Such a procedure, often called “relative dosimetry”, allows us to skip the time‐consuming production of a calibration curve for each irradiation. In this study, the magnitudes of errors due to the dose scaling procedure were evaluated by using the characteristic curves of BANG3 polymer gel dosimeter, radiographic EDR2 films, and GAFCHROMIC EBT2 films. Several sets of calibration data were obtained for each type of dosimeters, and a calibration equation of one set of data was used to estimate doses of the other dosimeters from different batches. The scaled doses were then compared with expected doses, which were obtained by using the true calibration equation specific to each batch. In general, the magnitude of errors increased with increasing deviation of the dose scaling factor from unity. Also, the errors strongly depended on the difference in the shape of the true and reference calibration curves. For example, for the BANG3 polymer gel, of which the characteristic curve can be approximated with a linear equation, the error for a batch requiring a dose scaling factor of 0.87 was larger than the errors for other batches requiring smaller magnitudes of dose scaling, or scaling factors of 0.93 or 1.02. The characteristic curves of EDR2 and EBT2 films required nonlinear equations. With those dosimeters, errors larger than 5% were commonly observed in the dose ranges of below 50% and above 150% of the normalization dose. In conclusion, the dose scaling for relative dosimetry introduces large errors in the measured doses when a large dose scaling is applied, and this procedure should be applied with special care.

PACS numbers: 87.56.Da, 06.20.Dk, 06.20.fb

## I. INTRODUCTION

A relative dosimetry technique is commonly used to validate dose distributions from treatment planning software in comparison to measured dose distributions. For the analysis, a physicist initially obtains calibration data (or characteristic curves or sensitometric data) that relate the signal from a dosimeter (or a detector) to the actual delivered dose. The calibration data are used to convert the detector signals to the absorbed doses to obtain a measured dose distribution. The response characteristic to radiation of a new batch of a detector is generally different from the dose response of the detector that is used for the initial collection of the calibration data. To accelerate the measurements, one usually skips taking a new set of calibration data and, instead, scales the measured doses to match those with an expected dose at a point, or in a small region, of a dose distribution. However, the validity of this procedure depends on the detector characteristics. In a previous work, we showed that a dosimeter with a linear calibration equation with zero dose‐offset enables us to perform precise relative dosimetry without calibration data. Furthermore, the linearity of the calibration equation justifies useful data manipulations, such as scaling the dose and changing the dose‐offset, for comparing dose distributions.^(1)^

In this study we evaluated the dosimetric errors associated with the dose scaling procedure. In the first section, we formulate a general theory of dose scaling as applied to relative dosimetry. It is followed by sections discussing the method we use to estimate the errors due to the dose scaling for dosimetric tools having both linear and nonlinear calibration equations. To demonstrate the procedure and to estimate the expected errors with actual dosimeters, we select three types of dosimeters, namely, BANG3 polymer gel dosimeter, radiographic EDR2 film, and GAFCHROMIC EBT2 film. With the polymer gel dosimeter, the errors are evaluated when the calibration curve of the polymer gel from a specific batch is used with three other batches of polymer gel. The EDR2 film exhibits small but not negligible energy dependence. Using the calibration data taken independently for three photon energies, we estimate the errors when the calibration curve of a specific energy is applied to the films exposed to other energies. When calibration data are repeatedly measured, the calibration curves show variation because of many factors including the scanning conditions. For EBT2 films, we obtain a set of calibration data with statistical deviation by collecting the data for several batches of films. The impact of the deviation of the calibration data on the dose estimation after the dose scaling is investigated. These examples are used to show a potential drawback associated with the dose scaling procedure commonly performed in a clinical setting, and the results should be able to inform medical physicists on the significance of expected errors.

## II. MATERIALS AND METHODS

### A. Theory

#### A.1 General theory

**Figure 1:**
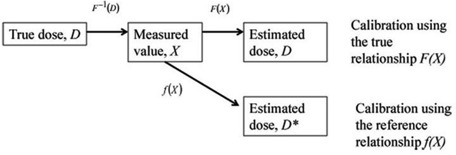
Illustration showing the dose scaling process.

For a radiation detector, we usually have a set of calibration data (or calibration curve) which relates a delivered dose *D* to the signal, *X*, recorded by the detector. The calibration data can be represented by a calibration equation (or “reference relationship” in Fig. 1):

(1) D=f(X)

Now, suppose we use the same type of the detector from a different batch. This detector may be associated with a calibration curve different from Eq.(1). We represent this true calibration equation (or “true relationship” in Fig. 1) by:

(2) D=F(X)

Note that the function *f* and *F* can be sometimes called a response function of a detector.

Now, let us consider a standard practice, in which we compare a measured dose distribution with a dose distribution calculated by treatment planning software. For this measurement, we use a detector — for example, a radiographic film — whose reference relationship *f*(*X*) is already known. Ideally, new calibration data or the true relationship *F*(*X*) should be obtained for this detector. For practical reasons, however, we often use the reference relationship *f*(*X*) of the detector, despite the expected difference in radiation response among different batches of the same type of detector. This procedure obviously results in incorrect dose measurements even if we otherwise use an accurate detector which can provide the exact dose when the true calibration equation is applied.

Generally, a relative dosimetry technique is used to remedy the aforementioned issue. For relative dosimetry, the measured dose is scaled to match the measured dose to the expected dose at one spatial point or a small area within a distributed dose. Suppose that the normalization dose, $D_{0}$, is delivered to a detector with a response function F. Then, the true measured detector signal $X_{0}$ is given by $F^{‐1}(D_{0}$) (as seen in Fig. 1). Since we do not know the true response function F, we use the reference response function *f*(*X*). Then, the measured estimated dose $D_{0}^{*}$ is obtained by $f(X_{0})$ for the given detector signal $X_{0}$ (see Fig. 1). Obviously, the estimated dose $D_{0}^{*}$ is not the true dose $D_{0}$. Let us introduce a “dose scaling factor” χ defined by:

(3) $$\chi =\frac{D_{0}}{D_{0}^{*}}=\frac{D_{0}}{f(X_{0})}$$

Now, consider a dose *D* different from $D_{0}$. By using the detector whose correct calibration curve is not known, the dose can be estimated by using the reference calibration data and the estimated dose is *D* ^*^, as seen from Fig. 1. To compensate the sensitivity difference, we now apply the dose scaling factor to all doses measured using this detector. It is easy to see that the scaled measured dose is equal to the dose scaling factor χ times the measured dose *D* ^*^. We introduce two definitions of errors, absolute error and relative error, to quantify the errors due to the dose scaling. The absolute error is the difference between the scaled dose and the true dose divided by the true dose. For the relative error, we take a ratio of the difference between the scaled dose and the true dose and a fixed “reference dose” $D_{r}$. The absolute and relative errors are denoted by ${ɛ}_{1}$ and ${ɛ}_{2}$, respectively, and the explicit formulas are given as follows:

(4) $$\varepsilon_{1}(D)=100\cdot \left(\frac{\chi D^{*}‐D}{D}\right)$$

(5) $$\varepsilon_{2}(D)=100\cdot \left(\frac{\chi D^{*}‐D}{D_{r}}\right)$$

Here $D_{r}$ is often the dose used for normalization (i.e., $D_{0}$). In fact, $D_{0}$ will always be used in the current study.

#### A.2 Linear relationship

In this section, we develop specific formulas of the errors given by Eqs.(4) and (5) when the calibration curve is given by a linear equation:

(6) D=aX+b

Let us represent the reference calibration equation by:

(7) $$D=a_{*}X+b_{*}$$

Suppose that we normalize the measured dose at $D_{0}$. When the calibration equation has no dose‐offset term, or $b=b_{*}=0$, the dose scaling factor can be expressed by:

(8) $$\chi_{0}=\frac{a}{a_{*}}$$

Using Eqs.(6) to (8) with Eq.(3), we can easily obtain the following expression of the dose scaling factor:

(9) $$\chi =\frac{\chi_{0}}{1+\frac{\chi_{0}\cdot b‐b_{*}}{D_{0}}}$$

Using Eqs.(6) to (9) with Eqs.(4) and (5), explicit formulas of absolute and relative errors can be derived as follows:

(10) $$\varepsilon_{1}(D)=100\left\{\frac{\chi }{\chi_{0}}\left(1+\frac{\chi_{0}\cdot b‐b_{*}}{D}\right)‐1\right\}$$

(11) $$\varepsilon_{2}(D)=100\left\{\left(\frac{\chi }{\chi_{0}}‐1\right)\frac{D}{D_{0}}+\frac{\chi }{\chi_{0}}\frac{\chi_{0}\cdot b‐b_{*}}{D_{0}}\right\}$$

Equation (9) indicates that $\chi =\chi_{0}$ when $b=b_{*}=0$. Then, it is easy to show from Eqs.(10) and (11) that both absolute and relative errors become zero. In other words, when the calibration curve can be represented by a linear equation without dose‐offset, the dose scaling procedure dose not introduce the error of the measured dose, although the true relationship of the specific batch of the detector is unknown.

Equations (10) and (11) show a clear difference between absolute and relative errors when both *b* and $b_{*}$ are not zero. We can easily identify some characteristics of the absolute error from Eq. (10). The magnitude of the absolute error increases infinitely as the dose decreases to zero. The absolute error monotonically changes with increasing dose. In particular, as the dose increases, it asymptotically approaches

(12) $$\underset{D\to \infty }{Lim}\varepsilon_{1}(D)=100\left(\frac{\chi }{\chi_{0}}‐1\right)$$

This equation implies that the asymptotic value of the absolute error is larger for a larger dose scaling factor. On the other hand, the relative error does not exhibit an asymptotic behavior with increasing dose. The relative error linearly changes with the dose, and it does not diverge at zero‐dose.

#### A.3 Nonlinear relationship

When the calibration equation is not linear, there is no simple analytical formula for the absolute and relative errors. Hence, we only discuss an algorithm to estimate the errors using Eqs.(4) and (5). First, suppose that for a type of a detector we know a reference relationship between dose *D* and the detector signal *X*, which is given by a function D=f(X). Now pick another detector (as denoted by A) from a different batch. Suppose that the true calibration equation of the detector A is D=F(X). To obtain the errors due to the dose scaling as a function of a given dose *D*, we use the following algorithm:

1) Select a dose *D*.
2) Obtain the signal of detector A, *X*, using the function *F* ^*‐1*^(*D*).
3) Calculate the dose to detector A by the reference relationship (i.e., D*=f(X)).
4) Obtain the dose scaling factor χ so that χD*=D at a normalization dose $D_{0}$, or by using Eq. (3).
5) Calculate the absolute and relative errors using Eqs.(4) and (5) for doses other than $D_{0}$.

### B. Examples

#### B.1 BANG3 polymer gel dosimeter

For the current study, the calibration data of BANG3 polymer gel (MGS Research Inc., Madison, CT) were taken from a publication.^(2)^ For the experiment, the polymer gel was irradiated for different dose levels of 0 to 12 Gy, using the 6 MV photon beams from a Varian Clinac 2300C/D linear accelerator (Varian Medical Systems, Palo Alto, CA). For MRI‐based polymer gel dosimetry, a relationship between the delivered dose, *D*, and the spin‐spin relaxation rate, *X*, is obtained. In a low‐dose range, the dose response of the polymer gel can be considered to be linear. For the BANG3 polymer gel dosimeter, hence, the calibration relationship can be expressed by a linear equation given by Eq. (6). The coefficients *a* and *b* from four different batches of polymer gels are given in Table 1. Using the calibration data of the first batch as the reference data, we calculated the errors for three other batches. The measured doses were scaled at the normalization dose of 5 Gy.

**Table 1:** Coefficients in Eq. (6) for BANG3 polymer gel experiments.

*Batch No*.	*a*	*b*
1	0.8157	−2.5465
2	0.8741	−2.3260
3	0.8475	−2.4856
4	0.7788	−2.3201

#### B.2 Radiographic EDR2 film

The energy dependence of dose response of EDR2 films (Carestream Health, Inc., Rochester, NY) is well known. For example, the optical density changes up to 5% when the photon energy increases from 6 MV to 15 MV.^(3)^ Here, we obtained calibration data of EDR2 films for 6 MV, 10 MV, and 18 MV photon beams (10 cm x 10 cm field size) of an Elekta Synergy linear accelerator (Elekta AB, Stockholm, Sweden). The films were irradiated to 13 dose steps from zero to 4 Gy by placing the film orthogonal to the beam axis in a solid water phantom at 10 cm depth with 100 cm source‐to‐surface distance (SSD). The films were scanned by using a Dosimetry PRO Advantage scanner (VIDAR Systems Corporation, Herndon, VA). The calibration data were used for film dosimetry with the RIT113 Radiation Therapy QA software (Radiological Imaging Technology, Colorado Springs, CO). It is noted that in the RIT113 software, the calibration data are stored as a relationship between the absorbed dose and the ADC (Analog‐to‐digital conversion) value that is proportional to the transmitted photon intensity. The data points showing the dose‐ADC value relationship were fitted by using third‐order polynomials or a logarithmic equation with additional third‐order polynomial terms. When the net optical density (netOD) instead of the ADC value was used, the regression equations were forced so that the netOD value was zero for an unirradiated film. The calibration equations were approximated by third‐order polynomials. Table 2 shows the coefficients of the calibration equations used for the analyses. The data of the 6 MV photon beam were used to obtain the reference calibration equation.

**Table 2:** Coefficients of calibration equations used to represent the calibration curves of EDR2 films. For the regression analysis, the ADC values were divided by $10^{‐3}$.

(a) To convert netOD to dose in Gy.					
	*3rd*	*2nd*	*1st*	*0th*	*R2*
6 MV	0.2109	−0.7726	2.5145	0.0	0.9999
10 MV	0.2016	−0.7731	2.4495	0.0	0.9998
18 MV	0.228	−0.8547	2.4629	0.0	0.9994

(b) To convert dose in Gy to netOD.					
	*3rd*	*2nd*	*1st*	*0th*	*R2*
6 MV	−0.0166	0.1140	0.3556	0.0	1.0000
10 MV	−0.0201	0.1363	0.3524	0.0	0.9999
18 MV	−0.0255	0.1651	0.3325	0.0	0.9995

(c) To convert ADC value to dose in Gy.					
	*Ln(x) Term*	*2nd*	*1st*	*0th*	*R2*
6 MV	−0.7483	−0.0002	0.0042	2.9909	NA
10 MV	−0.7104	−0.0003	0.0073	2.8303	NA
18 MV	−0.6904	−0.0004	0.0101	2.7556	NA

(d) To convert dose in Gy to ADC value.					
	*3rd*	*2nd*	*1st*	*0th*	*R2*
6 MV	−1.1501	11.197	−36.787	41.708	0.9999
10 MV	−1.2135	11.698	−37.804	41.746	0.9998
18 MV	−1.2129	11.743	−38.043	41.826	0.9998

#### B.3 GAFCHROMIC EBT2 film

GAFCHROMIC EBT2 films (Ashland Inc., Covington, KY) were irradiated with a 6 MV photon beam from Varian iX (Varian Medical Systems, Palo Alto, CA) for 12 dose steps varying from zero to 3.0 Gy. Seven sets of calibration data were obtained from five batches of EBT2 films, which were manufactured from June 2009 to August 2010. The films were scanned with an Epson 1000 flatbed scanner (Epson‐Seiko Co., Long Beach, CA). The film orientation was always set to the portrait (or coating) direction for the scanning. The scanned images were saved as TIFF (Tagged Image File Format) files. The scanned images were analyzed by using the OmniPro I'mRT software (IBA, Bartlett, IL), in which the measured signal values were saved on an inverted scale as ADC values. The mean and one standard deviation of ADC values from seven datasets were individually calculated for all 12 doses. Regression analysis was done using 5th or 6th order polynomial functions, of which coefficients are shown in Table 3. The regression equation of the mean ADC values was considered as the reference calibration equation. Two datasets obtained by the mean ADC values plus or minus one standard deviation were considered as the data, for which the true calibration equations were not known.

**Table 3:** Coefficients of polynomial equations used to represent the calibration curves of EBT2 films. For the regression analyses the ADC values were multiplied by $10^{‐4}$.

(a) To convert dose in Gy to ADC value								
	*6th*	*5th*	*4th*	*3rd*	*2nd*	*1st*	*0th*	*R2*
Average	−0.1326	1.3335	−5.2103	9.9077	−9.4059	4.7083	1.3219	0.9987
+SD	−0.0990	1.0154	−4.0642	7.9515	−7.8102	4.1657	1.4364	0.9986
−SD	−0.1663	1.6516	−6.3563	11.864	−11.002	5.2509	1.2074	0.9988

(b) To convert ADC value to dose in Gy.								
	*6th*	*5th*	*4th*	*3rd*	*2nd*	*1st*	*0th*	*R2*
Average	0	0.2905	‐3,4544	15.817	−34.216	35.239	−13.943	0.9990
+SD	0	0.2560	−3.1488	14.908	−33.354	35.569	−14.607	0.9991
−SD	0	0.3351	−3.8416	16.961	−35.349	35.007	−13.268	0.9987

#### B.4 Dose distributions of an IMRT plan

As a practical example, we investigated the effect of dose scaling for a head‐and‐neck IMRT case, which was planned to deliver 2 Gy per fraction using a 9‐field step‐and‐shoot technique with a 6 MV photon beam from Elekta Synergy linear accelerator. For QA, the gantry angles of all nine fields were set to zero. An EDR2 film was placed at a depth of 5 cm orthogonal to the beam axis direction in a 30 cm by 30 cm by 30 cm solid water phantom, which was set to 100 cm SSD. For absolute dose measurement, a Farmer‐type ionization chamber was inserted into the phantom at a depth of 6 cm along the beam axis. The dose distribution measured by EDR2 films were compared with that from the Pinnacle^3^ treatment planning software Version 9.0 (Philips Healthcare, Andover, MA) using the RIT13 Radiation Therapy QA software. The ADC data acquired by the RIT13 program were converted to dose by applying the calibration curves (i.e., ADC value vs. dose relationships) of 6 M V, 10 M V, and 18 MV photon energies. Note that those calibration data were the same as those discussed in Section II.B.2 above. Those three dose distributions were compared independently with the calculation using the gamma index, dose difference, and distance‐to‐agreement (DTA). The tolerance criteria for these analyses were set to 3% dose difference and 3 mm DTA. Note that the dose comparison was done by normalizing the measured dose to a calculated dose of 1.89 Gy averaged over a 2 mm by 2 mm area at the center of the distribution.

## III. RESULTS

### A. BANG3 polymer gel

**Figure 2:**
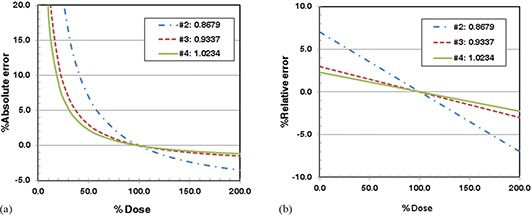
Percentage errors of estimated dose for BANG3 polymer gel example. (a) Absolute percentage error, (b) Relative percentage error.

The scaling factors obtained from Eq.(9) were 0.868, 0.934, and 1.023 for batch #2, #3, and #4, respectively (see Table 4). The absolute and relative errors were calculated using Eqs.(10) and (11). Those errors were plotted as a function of dose in (Figs. 2a) and (b). The absolute errors diverged as the dose decreases to zero as seen in (Fig. 2a). Using Eq. (12), we can show that the absolute errors asymptotically approached ‐6.99, ‐2.99, and ‐2.28 for batch #2, #3, and #4, respectively, for a large dose. The absolute errors were smaller than 2% between ‐40% and 200% of the normalization dose used for dose scaling (or 5 Gy) for batch #3 and #4. (Figure 2b) shows that the relative errors linearly changed with increasing dose. The relative errors were smaller than 2% in a wider dose range around the normalization dose than the absolute errors for batch #3 and #4. Batch #2 had the largest dose scaling factor (i.e., 13.2% difference from unity). The other two batches required smaller magnitude of scaling (i.e., 6.6% and 2.3% scaling for batch #3 and #4). (Figures 2a) and (b) clearly show that the errors are larger for a batch with a larger magnitude of dose scaling.

**Table 4:** Dose scaling factors used in example cases and corresponding average absolute and relative percentage errors.

				*Average Absolute Percentage Error [%]*			*Average Relative Percentage Error [%]*		
*Instrument*	*Batch Name*	*Dose Scaling Factor*	*Deviation from Unity [%]*	$<D_{0}$	$>D_{0}$	*Whole*	$<D_{0}$	$>D_{0}$	*Whole*
BANG3 polymer gel	#2	0.868	13.2	18.7	3.51	8.93	8.39	4.19	6.40
	#3	0.934	6.6	8.01	1.50	3.82	1.79	3.59	2.74
	#4	1.023	2.3	6.11	1.14	2.91	1.37	2.74	2.09
EDR2 film	10 MV (netOD)	0.955	4.5	6.00	0.86	3.96	0.77	1.62	0.97
	18 MV (netOD)	0.932	6.8	9.20	0.94	5.93	1.33	1.70	1.34
	10 MV (ADC)	0.940	6.0	4.04	5.90	5.61	1.64	9.80	1.64
	18 MV (ADC)	0.918	8.2	4.32	6.60	5.62	1.88	10.15	2.12
EBT2 film	+SD	0.945	5.5	10.02	1.41	6.76	1.71	1.91	1.64
	−SD	1.072	7.2	11.28	4.06	8.28	2.29	5.40	2.88

Notes: $D_{0}$ is the dose to which the dose was normalized. “Whole” means the average over the entire dose range.

To further quantify the effect of the dose scaling on the errors with estimated doses, we calculated both absolute and relative percentage errors by taking an average of the magnitude of the absolute and relative errors over three dose ranges: zero to the normalization dose, $D_{0}$, $D_{0}$ to maximum dose, and the entire range of zero to the maximum dose. The results for the BANG3 polymer gel case are shown in Table 4, which clearly shows that the average errors increased as the magnitude of dose scaling increased. For example, the average absolute and relative errors for the entire dose range were 8.93% and 6.40%, respectively, when larger than 10% of dose scaling is needed (as for batch#2).

### B. Radiographic EDR2 film

**Figure 3:**
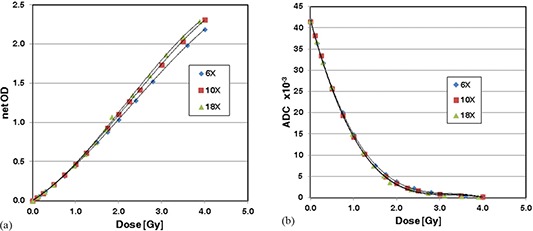
Calibration curves of EDR2 film for 6 MV, 10 MV, and 18 MV photon beams: (a) netOD vs. dose; (b) ADC value vs. dose.

The calibration data of EDR2 films using three photon energies (6 MV, 10 MV, and 18 MV) are presented in Fig. 3. The relationship between the dose and netOD is shown in (Fig. 3a); whereas (Fig. 3b) is for the dose versus ADC values. Also shown in the figures are regression curves. Note that for (Fig. 3a), the regression equations were derived so that netOD is zero for zero‐dose. The coefficients of determination are equal or close to unity for those regressions (see Table 3). The dose was normalized at 2 Gy.

**Figure 4:**
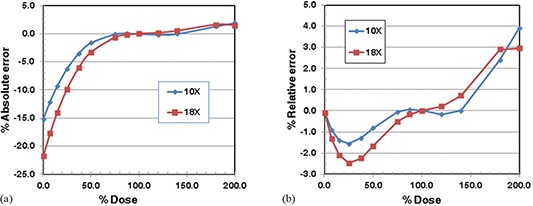
Percentage errors of estimated dose for EDR2 film example (NetOD was used): (a) absolute percentage error; (b) relative percentage error.

**Figure 5:**
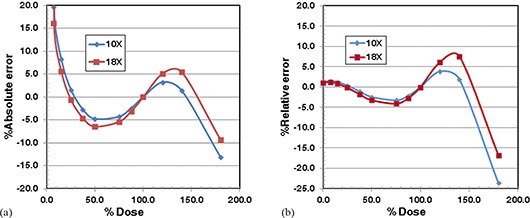
Percentage errors of estimated dose for EDR2 film example (the ADC values of photon transmission were used): (a) absolute percentage error; (b) relative percentage error.

First we present the results when netOD was used to represent the calibration data. (Figures 4a) and (b) show the absolute and relative errors when the data of a 6 MV photon beam were used as the reference calibration equation to convert netOD to doses for the data of 10 MV and 18 MV photon beams. The absolute error decreased from ‐15% or ‐22% at zero‐dose to ‐2% or ‐4% at the 50% dose level for 10 MV and 18 MV. The absolute errors were within ±3% in the dose level of 50% to 200%. The relative errors were within ±3% for 10 MV and 18 MV in the entire dose range, except above 180% dose level. Table 4 shows that the scaling factors for 10 MV and 18 MV were 0.955 and 0.932, respectively. The 18 MV data required larger dose scaling than 10 M V. The relative errors and to lesser extent the absolute errors shown in Fig. 4 indicates that the errors are larger for 18 MV than 10 MV. As seen in Table 4, the average absolute and relative percentage errors for the 18 MV photon beam were larger than those of the 10 MV photon beam because a larger dose scaling was needed with the 18 MV photon beam than with the 10 MV photon beam.

(Figures 5a) and (b) show the absolute and relative errors when the ADC value versus dose relationship was used. The absolute errors were larger than 10% for dose below 10% dose level. Between 10% and 150% dose level, the errors varied between ±7%. The relative errors did not show the singular behavior near the zero‐dose. The relative errors were also within ±5% up to 120% dose level for both 10 MV and 18 MV. (Figures 5a) and (b) indicate that the errors

of the 10 MV photon beam with the scaling factor of 0.940 are smaller than the errors of the 18 MV photon beam with the scaling factor of 0.918 for doses lower than 140% dose. Such a dependence of the errors on the dose scaling factor was more clearly seen in the average absolute and relative percentage errors shown in Table 4. The average absolute and relative percentage errors were 1.62%/2.12% and 5.9%/6.6% for 10 MV and 18 MV, respectively.

### C. GAFCHROMIC EBT2 film

**Figure 6:**
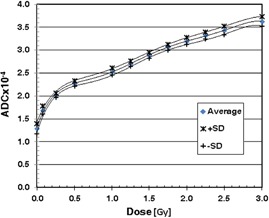
Calibration curves of EBT2 film.

**Figure 7:**
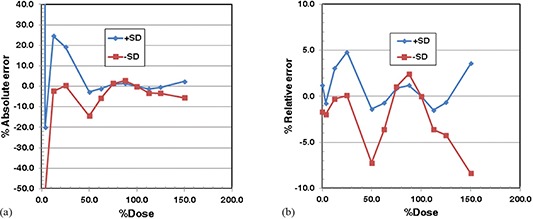
Percentage errors of estimated dose for EBT2 film example: (a) absolute percentage error; (b) relative percentage error.

The dose‐to‐ADC value relationships are shown for the mean ADC values and the ADC values plus or minus one standard deviation (+SD and −SD) in Fig. 6. Note that the standard deviation of the ADC values decreased with increasing dose (i.e., 8.6% for zero‐dose to 2.8% at 3.0 Gy). The ADC values show a sharp decrease near the zero‐dose. Because of this behavior, high order (or 6th order) polynomial functions were required for regression. (Figures 7a) and (b) show the absolute and relative errors. The magnitude of the absolute errors was larger than 10% for dose below 50% of the normalization dose (i.e., 2.0 Gy) for the −SD data and for dose below 30% for the +SD data. The absolute errors were within ±5% for dose between 60% and 130% dose level. The relative errors were within ±5% for dose up to 150% dose level, except at 50% dose and above 130% dose for the −SD data. In the dose ranging from 70% to 120% of the normalization dose, both absolute and relative errors were smaller than 4%. The average absolute and relative percentage errors for the entire dose range were 6.76% and 1.64%, 8.28% and 2.88% for the +SD and −SD cases, as seen in Table 4. The −SD case needed a larger dose scaling than the +SD case (i.e., 7.2% vs. 5.5%); hence, the errors of the −SD case were larger than those of the +SD case.

### D. Dose distributions of an IMRT plan

The results of dose comparison between the EDR2 film measurement and the Pinnacle calculation using three calibration data were presented in Table 5. The absolute dose measured with an ionization chamber differed from the calculated dose only by 0.4%. The radiation sensitivity of EDR2 films used for this measurement was different from that of the calibration films by about 1 % at 2 Gy. For the comparison, we used dose normalization factors (or dose scaling factors) of 0.988, 0.944, and 0.921 for the 6 MV, 10 M V, and 18 MV calibration data, respectively. The dose scaling factors decreased as the calibration energy increased, as expected from the results presented in Section III.B above. The passing rates of gamma index were 95.8%, 94.7%, and 87.9%, for the 6 MV, 10 MV, and 18 MV data. The statistics of the dose difference and DTA passing rates showed a similar trend with increasing photon energy or increasing magnitude of dose scaling, as seen in Table 5.

**Table 5:** Dosimetric errors introduced by using the calibration data of 10 MV and 18 MV for the dose distribution analysis of 6 MV photon beam IMRT treatment. The tolerance values were 3% dose difference and 3 mm distance‐to‐agreement (DTA).

*Photon Energy*	*Dose Scaling factor*	*Gamma Index*	*Passing Rate [%] Dose Difference*	*DTA*
6 MV (energy)	0.988 (1.000)^a^	95.8	81.9	79.9
10 MV	0.944 (0.956)	94.7	80.9	77.8
18 MV	0.921 (0.932)	87.9	73.3	61.8

a The values in parentheses indicate the dose scaling factors relative to that of 6 MV.

## IV. DISCUSSION

The magnitude of the dose scaling affects the errors in the estimated measured doses. The larger the dose scaling the larger the errors are. This effect is clearly seen when the calibration data can be represented by a linear equation such as for BANG3 polymer gel dosimeter. However, such a trend is not as clear as the linear case when the calibration equation is nonlinear, unless the errors averaged over a dose range are used. It is noteworthy that the errors also vary largely due to the difference in the shape of the calibration curves.

The current results show generally that the dosimetric error could exceed 5% in the clinically important dose range (i.e., 20% to 120% of the normalization dose) when the dose scaling is more than 5%. If the dose scaling is larger than 10%, the error is larger than 5% even inside the clinically important dose range. Therefore, we recommend an additional investigation on the cause of the required large scaling if the dose scaling factor were different from unity by more than 5%.

There is a trend in the medical physics community toward a use of the direct photon transmission values (or the ADC values) instead of netOD to represent a calibration data for film dosimetry. However, our study using the EDR2 film data demonstrated a possibility to decrease the errors due to dose scaling by using netOD.

The EDR2 film example simulated realistic cases, for which a calibration data of single energy is applied to the films irradiated with multiple energy beams. Such cases commonly occur in practice.^(4)^ Hence, physicists should be aware of potential errors when they do relative dosimetry.

The IMRT example using EDR2 films demonstrated that increasing magnitude of dose scaling resulted in increasing dosimetric errors when dosimetric parameters, such as the passing rate of gamma index that is routinely used under real clinical settings, were evaluated. This example used three calibration curves taken for three different energies. But, the result should be true even if the calibration data of the single energy is used, since the difference among those three calibration curves are similar to the expected film‐to‐film variation of radiation sensitivity among EDR 2 films. In fact, Childress et al.^(5)^ did a comprehensive study showing the film‐to‐film variation of EDR2 film sensitivity. Their results indicate that the standard deviation in the optical density at dose greater than 1 Gy is 8%.

The example of EBT2 films showed a potential variation of the calibration equation by considering the range of measured signal within one standard deviation. Such a variation is caused by the film sensitivity variation among the different batches. Our results showed that the inter‐batch variation of the sensitivity can lead to easily 5% or larger error when the dose scaling is applied to the measured doses. On the other hand, the intra‐batch (or film‐to‐film) variation of the film sensitivity is smaller than 1%, and the uncertainty in the optical density is about 1%.^6^, ^9^ Hence, the errors with dose scaling procedure are smaller than those expected for the inter‐batch variation, but a potentially large error should be anticipated.

In this work, the errors due to the dose scaling procedure were evaluated using two definitions, namely the absolute error and the relative error. The absolute error is in a sense a true error, because the difference between the scaled measured dose and the true dose is divided by the true dose, whereas the relative error was calculated by dividing the dose difference by a reference dose such as the normalization dose. Consequently, the absolute error is larger than the relative error; in particular, the absolute error diverges as the dose decreases to zero.

A previous publication^(1)^ showed a difficulty of high‐precision relative dosimetry if the calibration relationship between the dose and the detector signal is either linear with dose‐offset or nonlinear. The current study showed that the magnitude of errors associated with dose scaling procedures easily exceeds 5% by using realistic examples for three dosimeters, two of which are commonly used for clinical dosimetry.

## V. CONCLUSIONS

Dose scaling procedure introduced large dosimetric errors, particularly for doses much lower or larger than the normalization dose used for the dose scaling. The errors increase with increasing magnitude of dose scaling. Hence, if the dose scaling factor is different from unity — generally, 5 % or larger — the dose comparison should be carefully evaluated for the accuracy of the results. It should be noted also that the absolute error can be very large for doses much lower than the normalization dose.
